# Involvement of premacular mast cells in the pathogenesis of macular diseases

**DOI:** 10.1371/journal.pone.0211438

**Published:** 2019-02-22

**Authors:** Takaki Sato, Seita Morishita, Taeko Horie, Masanori Fukumoto, Teruyo Kida, Hidehiro Oku, Kimitoshi Nakamura, Shinji Takai, Denan Jin, Tsunehiko Ikeda

**Affiliations:** 1 Department of Ophthalmology, Osaka Medical College, Takatsuki-City, Osaka, Japan; 2 Nakamura Eye Clinic, Matsumoto-City, Nagano, Japan; 3 Department of Innovative Medicine, Graduate School of Medicine, Osaka Medical College, Takatsuki-City, Osaka, Japan; Faculty of Medicine & Health Science, UNITED ARAB EMIRATES

## Abstract

We previously reported on the elevated intravitreal activities of tryptase and chymase in association with idiopathic epiretinal membrane (ERM) and idiopathic macular hole (MH). In this present study, we investigated the potential intraocular production of these serine proteases, and measured and compared tryptase and chymase activities in the vitreous body and serum in ERM, MH, proliferative diabetic retinopathy (PDR), and rhegmatogenous retinal detachment (RRD) patients. In addition, nuclear staining with hematoxylin and eosin (H&E) and mast-cell staining with toluidine blue were performed on samples of the vitreous core and bursa premacularis (BPM) of MH. We also performed immunostaining on the above two regions of vitreous samples for MH with anti-tryptase antibody, anti-chymase antibody, anti-podoplanin antibody, anti-lymphatic vessel endothelial hyaluronan receptor 1 (LYVE-1) antibody, and anti-fibroblast antibody. Moreover, we performed immunostaining with anti-tryptase antibody and anti-chymase antibody on ERMs collected intraoperatively. Tryptase activity in the vitreous body was significantly higher in ERM and MH than in PDR. However, no significant differences were observed in the tryptase activity in the serum among these four diseases. Chymase activity in the vitreous body was significantly higher in MH than in the other three diseases, yet chymase activity in the serum was below detection limit in any of the diseases. Nuclear staining with H&E revealed an abundance of nuclei in the BPM region, but few in the surrounding area. Mast-cell staining with toluidine blue revealed that the BPM showed metachromatic staining. In immunostaining with anti-fibroblasts antibody, anti-tryptase antibody, anti-chymase antibody, anti-podoplanin antibody, and anti-LYVE-1 antibody, the BPM stained more strongly than the vitreous core. Tryptase and chymase-positive cells were also observed in ERM. These findings revealed that the presence of mast cells in the BPM potentially represent the source of these serine proteases. Moreover, the BPM, as a lymphatic tissue, may play an important role in the pathogenesis of macular disease.

## Introduction

Idiopathic epiretinal membrane (ERM) and idiopathic macular hole (MH) are known to cause metamorphopsia and reduced visual acuity, and occur mainly in middle-aged and older adults. At present, there are no effective pharmacotherapies for ERM and MH, except ocriplasmin for MH [1]. Thus, vitreous surgery is the primary therapeutic option. It has been proposed that the causes of ERM include a mechanism of vitreous traction on the macula that initiates cell proliferation or extracellular matrix accumulation on the posterior wall of the posterior precortical vitreous pocket (PPVP) [2], an anatomical structure previously termed 'bursa premacularis' (BPM) by Worst in 1977 [3], and that vitreomacular traction is also generally accepted as a cause of MH [4]. It has been considered that the PPVP and the BPM are arguably the same space. Besides the thin membrane remaining on the retina after artificial posterior vitreous detachment has been regarded as the posterior wall of PPVP. However Polak et al injected TA inside the premacular thin membranous tissue, and demonstrated that the membranous tissue, itself, was the BPM and the connecting cisternal system, i.e., the corona petaliformis of Worst, which encircled the BPM [5]. Fine and Spaide, as well as Sato et al, reportedly observed a similar phenomena [6, 7].Recently, several studies have investigated the morphology of ERM and MH using optical coherence tomography (OCT) [8, 9], however, few studies have been conducted to investigate biochemical features of these macular diseases.

In previous studies, we reported our findings in regard to the elevated activities of serine proteases in the vitreous of ERM and MH, including tryptase and chymase, and discussed their relationships to the pathogenesis of these diseases [10, 11]. In this present study, we used various clinical samples to investigate the differences in serine protease activities among different vitreoretinal diseases in order to elucidate the source of such proteases. In addition, immunohistochemical analysis of the premacular membrane was performed in order to confirm the nature and characteristics of the BPM investigate the properties of this interesting tissue.

## Subjects and methods

### Serine proteases activities in the vitreous body and serum in four vitreoretinal diseases

In this study, we examined and evaluated vitreous samples obtained from ERM, MH, proliferative diabetic retinopathy (PDR), and rhegmatogenous retinal detachment (RRD) patients, and measured tryptase activity in 20 patient eyes and chymase activity in 10 patient eyes. This study was approved by the Ethics Committee of Osaka Medical College (Approval No. 1134), and was performed in accordance with the tenets set forth in the Declaration of Helsinki. Informed written consent was obtained from all subjects prior to the preoperative blood test examination and the vitrectomy surgery being performed.

The method used for collecting the vitreous samples to measure serine proteases activities was as follows. Briefly, using a 25-guage (G) vitreous surgery system, trocars were placed at two locations, and vitreous gel in the vitreous core was collected with a vitreous cutter prior to perfusion. Serum samples were also collected from the patients at the same time. All obtained samples were then stored in a deep freezer, and subsequently measured for tryptase and chymase activities using a spectrophotometer (BioSpectrometer; Eppendorf AG, Hamburg, Germany).

Tryptase activities were measured via the cleavage of t-butyloxycarbonyl (Boc)-Phe-Ser-Arg-4-methylcoumarin-7-amide (MCA). Briefly, the vitreous samples were incubated at 25°C with 1 mM of Boc-Phe-Ser-Arg-MCA in 0.1 M of Tris-HCI buffer (pH 8.0) containing 1.8 M of KCI. Reactions were terminated by adding 3% metaphosphoric acid. MCA released from the substrate was then determined fluorimetrically at 370-nm excitation and 460-nm emission. One unit of tryptase activity was defined as the amount of enzyme that cleaved 1 μmol of MCA/minute.

Chymase activities were measured by incubating the vitreous samples at 37°C with 4 mM angiotensin I in 150 mM borax-borate buffer (pH 8.5) containing 8 mM dipyridyl, 770 μM di-isopropyl fluorophosphate, and 5 mM ethylenediaminetetraacetic acid. The reaction was then terminated by adding 15% trichloroacetic acid. One unit of chymase activity was defined as the amount of enzyme that cleaved 1 μmol of angiotensin II/minute.

### Nuclear staining with hematoxylin and eosin (H&E) and cytoplasmic staining with toluidine blue

**Fig 1:**
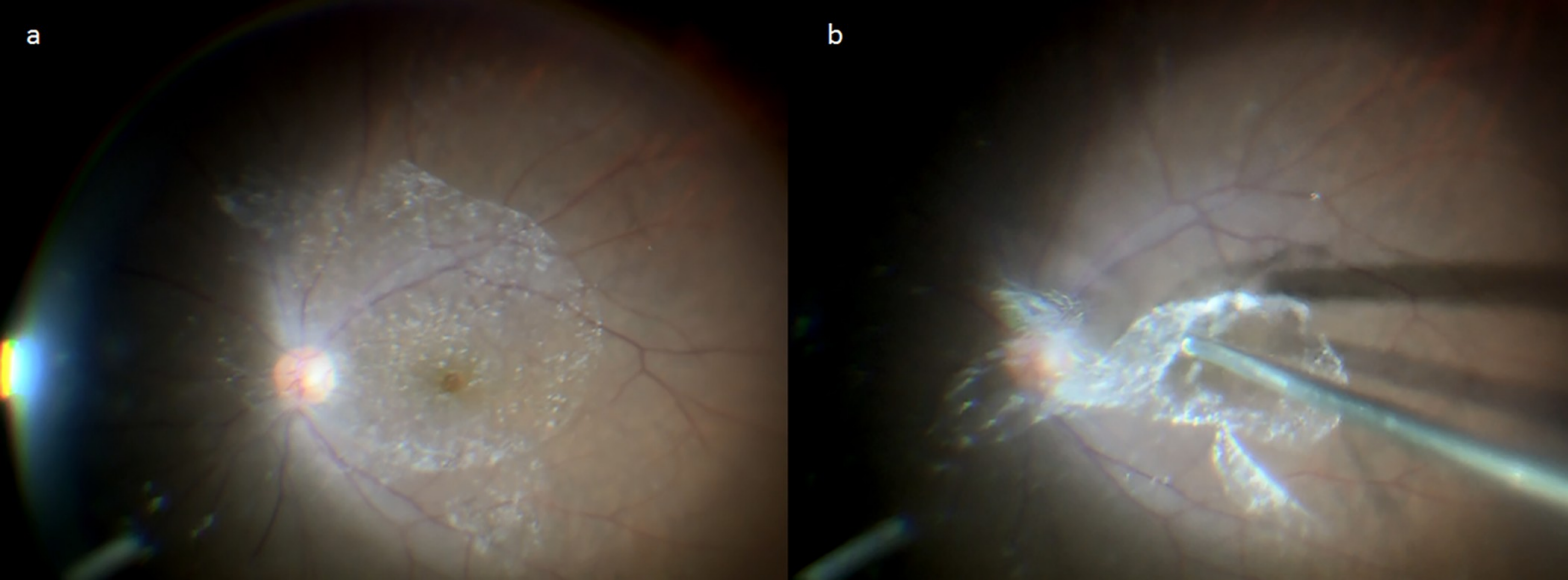
Method used for collection of the bursa premacularis (BPM). The BPM was visualized with an application of triamcinolone acetonide (TA) to the posterior pole of the vitreous (a). TA accumulated on the anterior surface of the BPM. A diamond-dusted membrane scraper was then used to form a window on a part of the BPM, from which the BPM was separated from the retinal surface by aspiration with a vitreous cutter to be selectively collected (b).

From 2 eyes of 2 patients with MH (a 70-year-old male and a 54-year-old female) accompanied with a clearly identifiable BPM on the surface of the macula, the vitreous core and the BPM were focally collected during vitreous surgery. The vitreous core samples were collected in the same surgical procedure as described above. BPM samples were collected in the following manner. Briefly, the anterior surface of the BPM was visualized with the application of TA to the posterior pole following extensive resection of the vitreous body (Fig 1A). A diamond-dusted membrane scraper was then used to form a window on a part of the BPM, from which the BPM was separated from the retinal surface by aspiration with a vitreous cutter. At the same time, the area of Martegiani of at the posterior end of Cloquet’s canal was also dissociated from the optic disc adhesion. Subsequently, the perfusate in the tube connected to the vitreous cutter was completely removed by a reverse thrust of air from the connector. The cutter was then reinserted into the vitreous body to selectively collect the BPM floating in the vitreous cavity (Fig 1B).

Samples of the vitreous core and BPM were fixed in 4% paraformaldehyde (PFA) after collection, and then rinsed with phosphate-buffered saline (PBS) (pH7.4). The 2 eyes underwent nuclear staining with H&E (Vector Laboratories, Inc., Burlingame, CA) and mast-cell staining with 0.05% toluidine blue (pH4.1) (Muto Pure Chemicals, Co., Ltd., Tokyo, Japan). The 2 eyes were then rinsed several times in PBS, dehydrated through a graded series of ethanol and xylene, mounted with Fisher Chemical Permount (Fisher Scientific, Waltham, MA), and observed under a fluorescence microscope (BZ-X700; Keyence Corporation, Osaka, Japan).

### Immunofluorostaining of the vitreous core and BPM

From 8 eyes of 7 patients with MH (2 males and 5 females, mean age: 67.2 years, age range: 62–77 years) with a clearly identifiable BPM, the vitreous core and BPM were focally collected during vitreous surgery. The methods used to collect the vitreous body samples were the same as described above.

Samples of the vitreous core and BPM were fixed in 4% PFA after collection and rinsed with PBS, followed by blocking treatment with 5% normal goat serum (NGS). Immunostaining was performed with the respective target antibodies via the avidin-biotin-peroxidase complex staining method. Of the 8 vitreous core and BPM samples, 2 were incubated with anti-tryptase antibody (rabbit polyclonal, 1:500; Abcam plc., Cambridge, MA), 3 were incubated with anti-chymase antibody (rabbit polyclonal, 1:500; Abcam), 1 was incubated with anti-podoplanin antibody (rabbit monoclonal, 1:500; Abcam), 1 was incubated with anti-lymphatic vessel endothelial hyaluronan receptor 1 (LYVE-1) antibody (rabbit polyclonal, 1:500; Abcam), and 1 was incubated with anti-reticular fibroblasts and reticular fibers antibody (ER-TR7, rat monoclonal,1:500; Abcam), all at 4°C for 2 days. The samples were then rinsed with PBS, and incubated with biotinylated anti-rabbit immunoglobulin G (IgG) (1:1000; Vector Laboratories) at room temperature (RT) for 2 hours. The samples were then rinsed again with PBS and incubated with alkaline phosphatase-labeled avidin-biotin complex (Vector Laboratories, Burlingame, CA) at RT for 2 hours, followed by reaction with ImmPACT Vector Red (Vector Laboratories). The samples were then dried, mounted with Entellan (Merck KGaA, Darmstadt, Germany), and observed under a fluorescence microscope (BZ-X700).

### Immunofluorostaining of ERM

ERM specimens were collected from 4 ERM patients (3 males and 1 female; mean age: 69.5 years; age range: 64–75 years). In each patient, membrane peeling was performed during vitreous surgery, and the ERM was gripped with vitreous forceps and removed from the trocar in order to collect the sample. The collected ERMs were then fixed in 4% PFA and rinsed with PBS. After blocking with 1% NGS or 1% normal donkey serum (NDS) plus 1.0% BSA and 0.1% triton X 100 in PBS, the ERM samples were incubated with anti-tryptase antibody (mouse monoclonal, 1:200; Santa Cruz Biotechnology, Inc., Dallas, TX) and anti-chymase antibody (goat polyclonal, 1:200; Santa Cruz Biotechnology) for 2 days at 4°C. The ERMs were then incubated at RT for 2 hours in Alexa 594 or Alexa 488-conjugated with the appropriate secondary antibodies (1:500; Invitrogen Corporation, Carlsbad, CA). An ERM was also counterstained with 4',6-diamidino-2-phenylindole (DAPI; Dojindo Molecular Technologies, Inc., Kumamoto, Japan), and then photographed with a fluorescence microscope (BZ-X700).

## Results

### Serine protease activities in the vitreous body and serum in four vitreoretinal diseases

**Fig 2:**
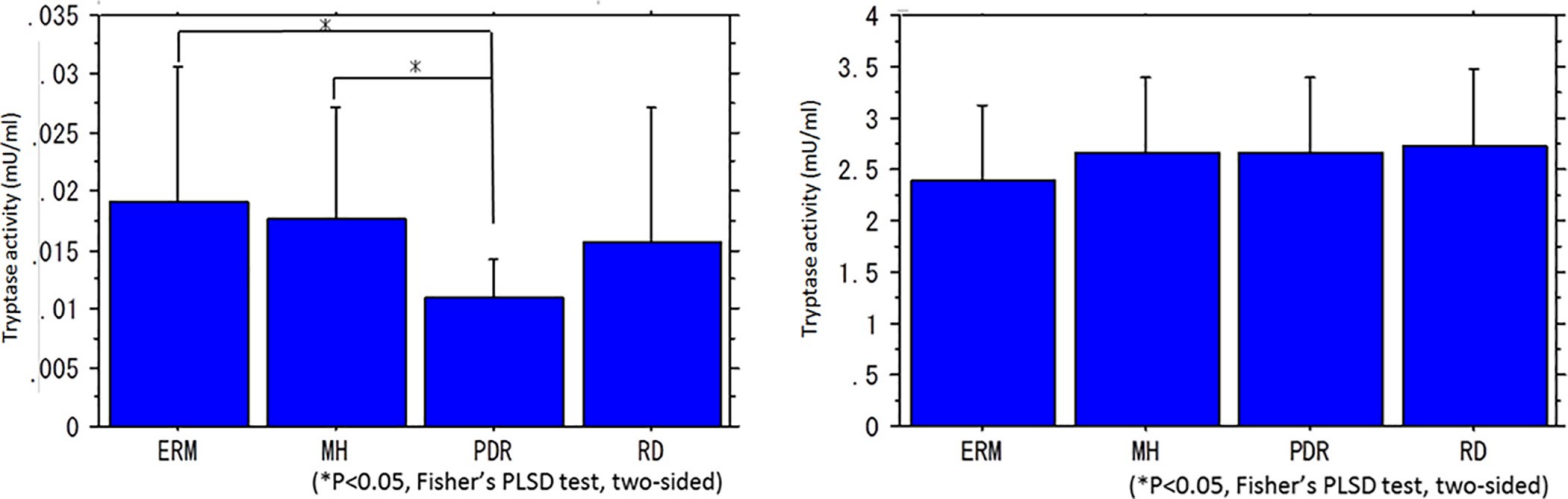
Tryptase activity in the vitreous body and serum. The levels of tryptase activity were significantly higher in ERM and MH than in PDR (a). No significant differences in tryptase activity were found in the serum obtained from the four diseases (b).

**Fig 3:**
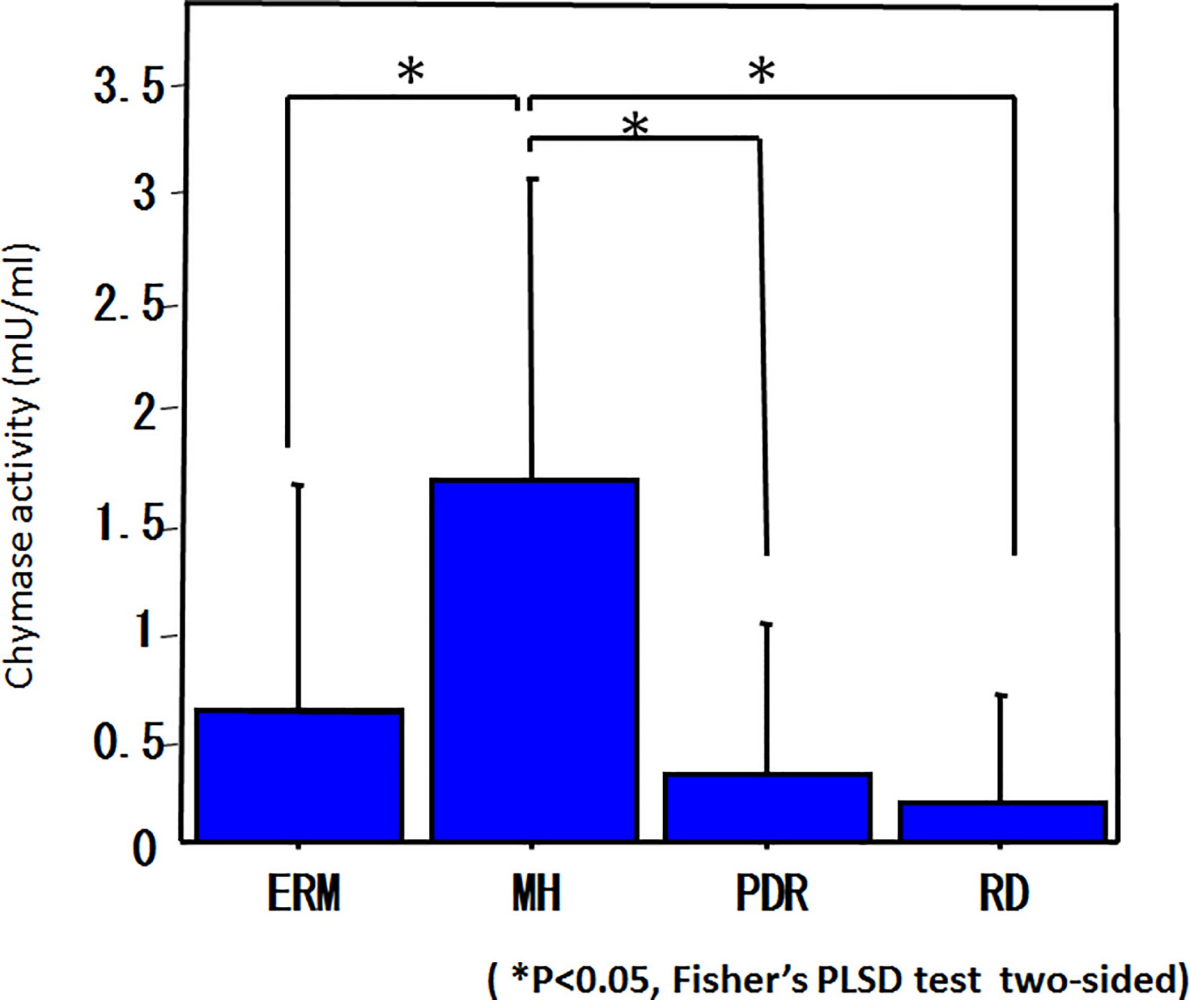
Chymase activity in the vitreous body and serum. The levels of chymase activity were significantly higher in MH than in ERM, PDR, or RRD. Chymase activity in the serum was below detection limit in any of the diseases.

The mean tryptase activity (mU/ml) in the vitreous body was 0.019 ± 0.011 in ERM, 0.018 ± 0.009 in MH, 0.011 ± 0.003 in PDR, and 0.016 ± 0.012 in RRD, showing a significantly higher level in ERM and MH than in PDR (Fig 2A). The mean tryptase activity in serum was 2.390 ± 0.736 in ERM, 2.665 ± 0.736 in MH, 2.664 ± 0.738 in PDR, and 2.719 ± 0.760 in RRD, thus showing no significant differences among the four diseases (Fig 2B). The mean chymase activity (mU/ml) in the vitreous body was 0.607 ± 1.035 in ERM, 1.651 ± 1.627 in MH, 0.312 ± 0.686 in PDR, and 0.178 ± 0.498 in RRD, showing a significantly higher level in MH than in ERM, PDR, or RRD (Fig 3). Chymase activity in the serum did not exceed the detection limit in any of the diseases.

### Nuclear staining with H&E and cytoplasmic staining with toluidine blue

**Fig 4:**
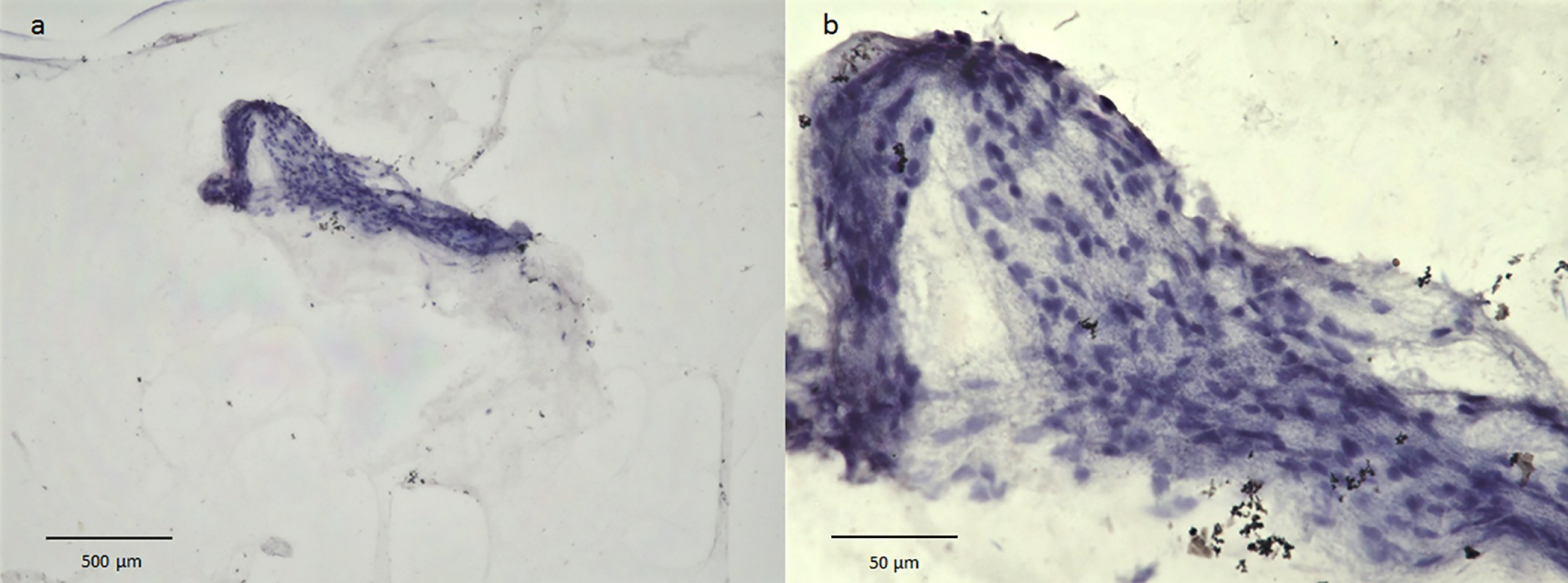
Nuclear staining with H&E. Nuclear staining with H&E reveals an abundance of nuclei in the BPM region, yet few in the surrounding area.

**Fig 5:**
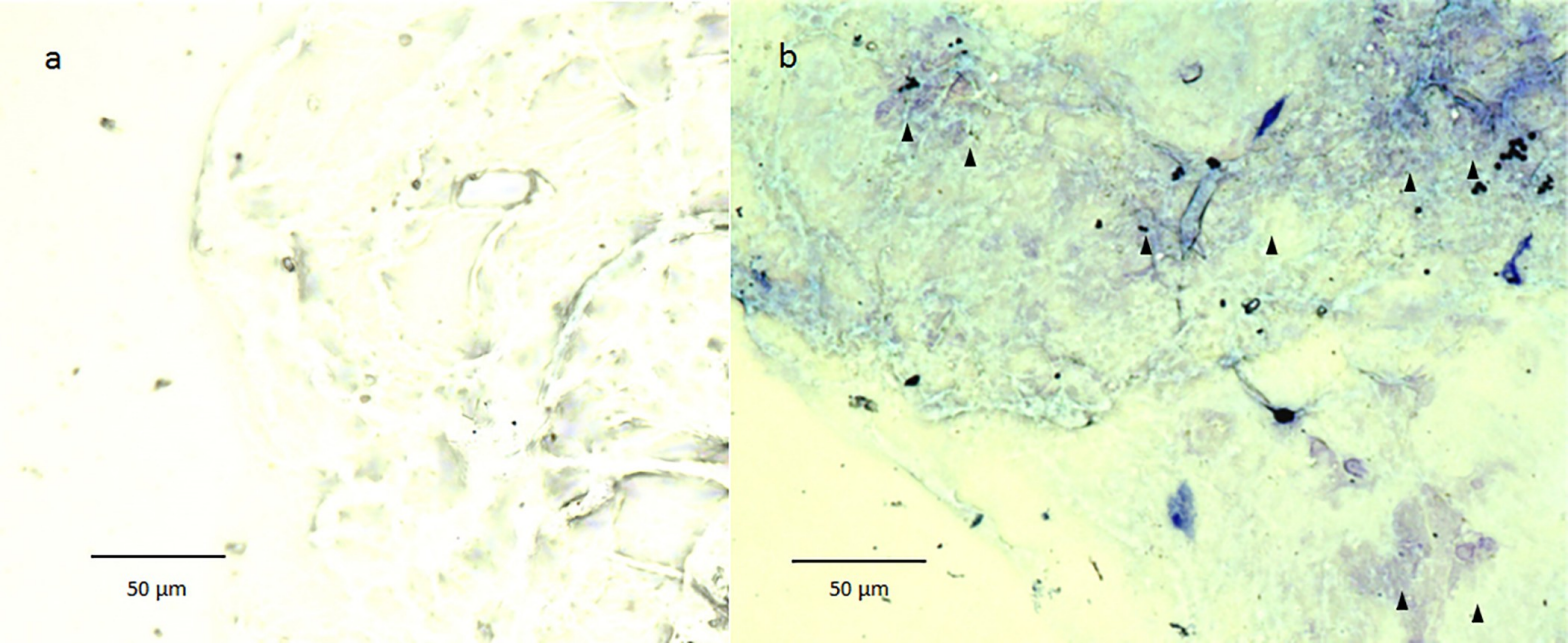
Cytoplasmic staining with toluidine blue. The vitreous core is weakly stained (a), however, the BPM shows intense and somewhat metachromatic staining (b: black arrowheads).

Nuclear staining with H&E revealed an abundance of nuclei in the BPM region, yet few in the surrounding area (Fig 4A and 4B). Cytoplasmic staining with toluidine blue revealed weak staining of the vitreous core, however, the BPM showed intense and somewhat metachromatic staining, thus indicating the presence of mast cells (Fig 5A and 5B).

### Immunofluorostaining of the vitreous core and BPM

**Fig 6:**
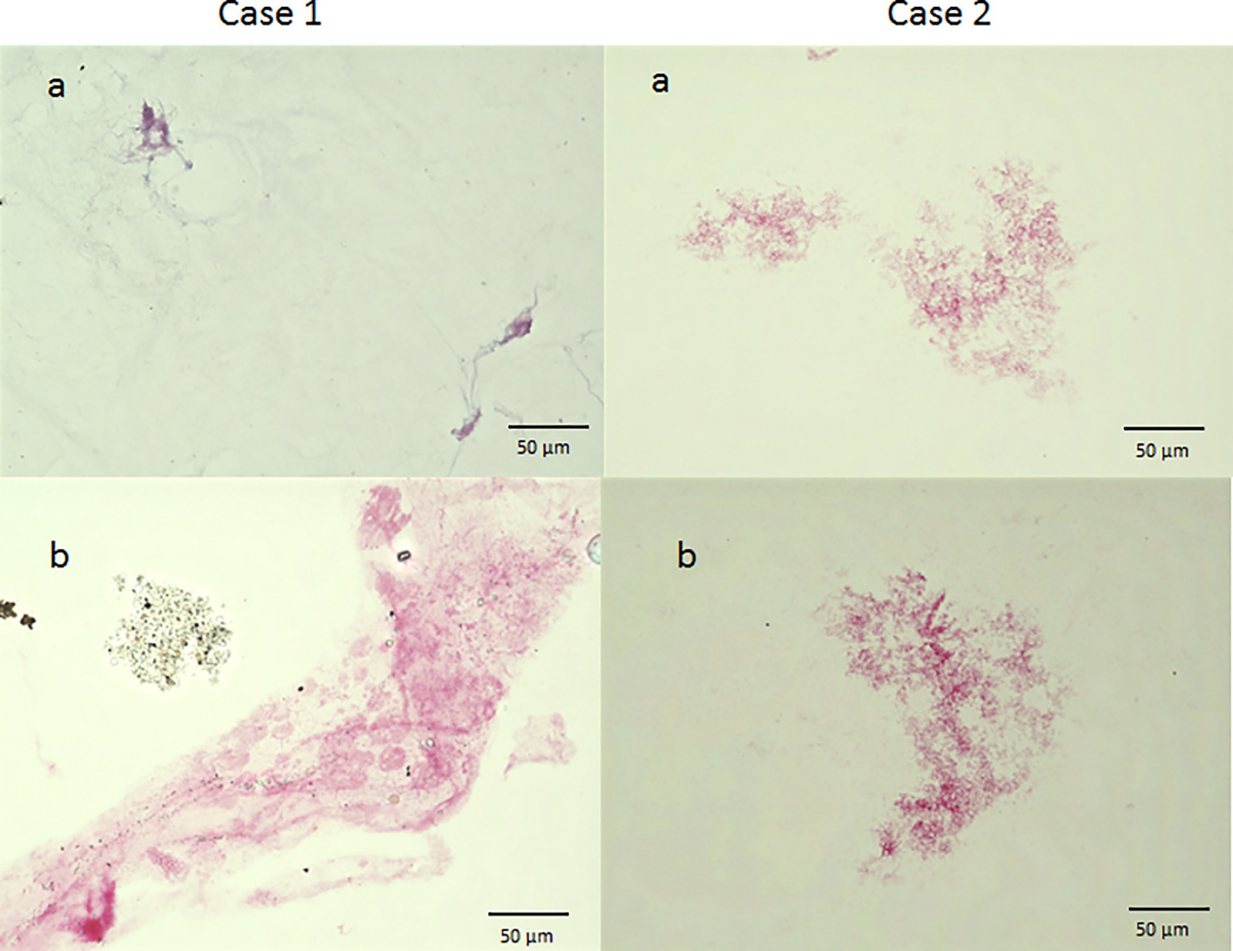
Immunostaining with anti-tryptase antibody. In both cases, the vitreous core is nearly unstained (a), yet the BPM region is stained, although weakly (b).

**Fig 7:**
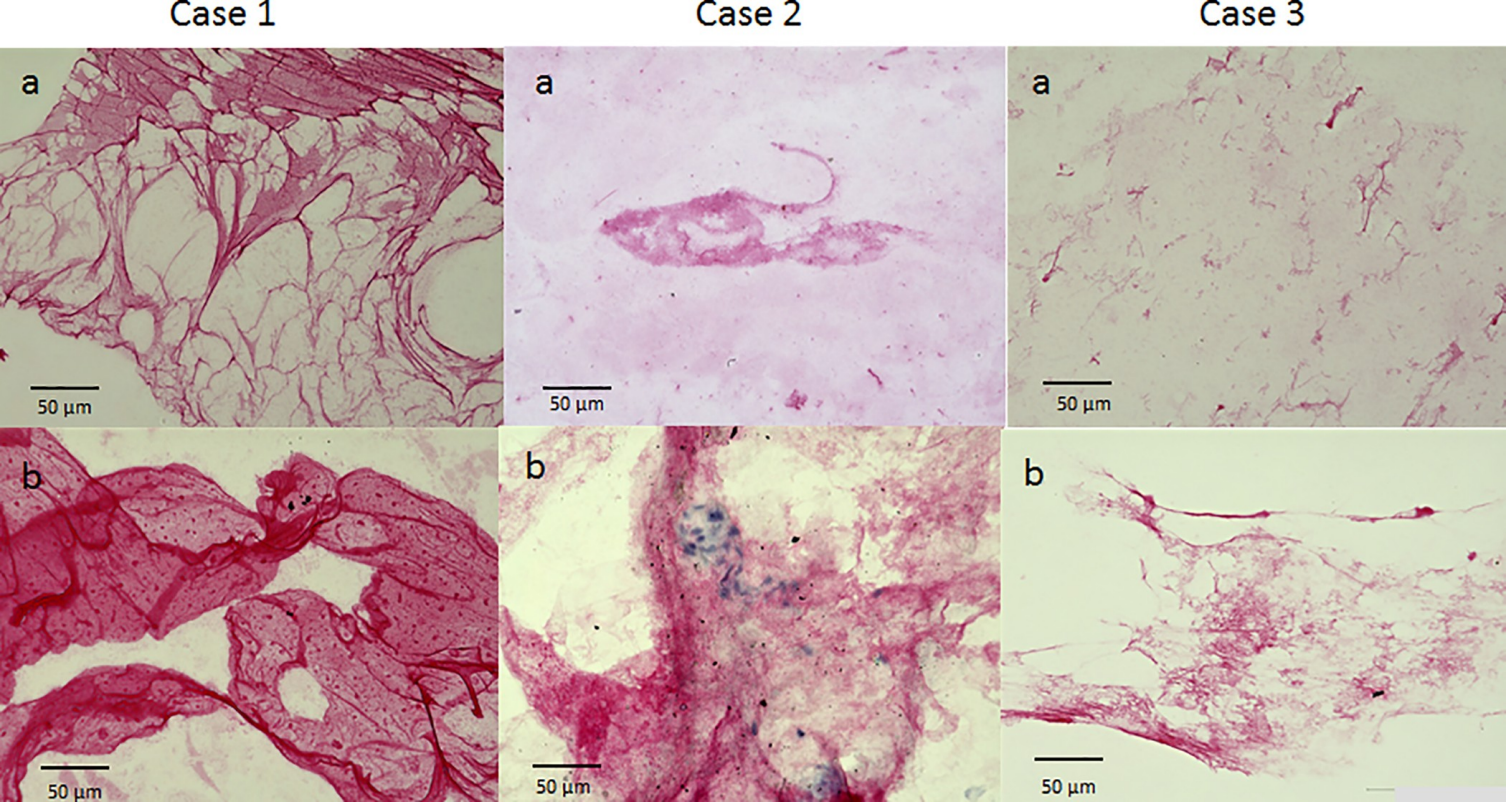
Immunostaining with anti-chymase antibody. In all 3 cases, the vitreous core is weakly stained (a), but the BPM is strongly stained (b). Moreover, in Case 1 and Case 2, the BPM region shows a membranous structure that includes numerous chymase-positive cells.

**Fig 8:**
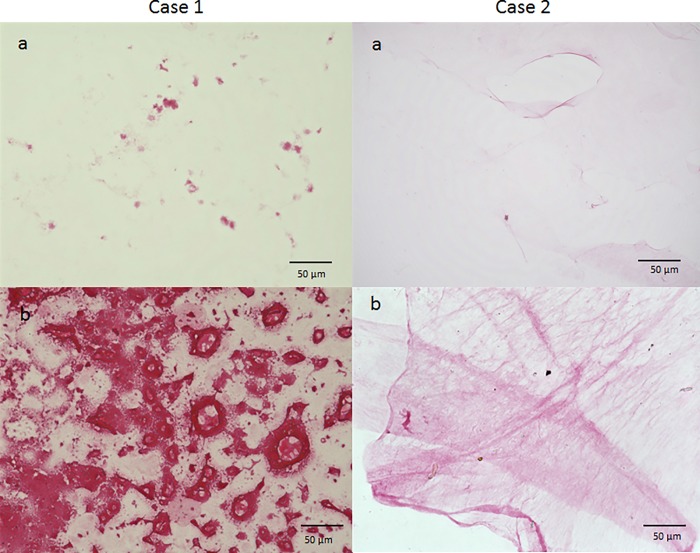
Immunostaining with anti-podoplanin antibody (Case 1) and anti-LYVE-1 antibody (Case 2). The vitreous core is weakly stained (a), yet the BPM is strongly stained (b).

**Fig 9:**
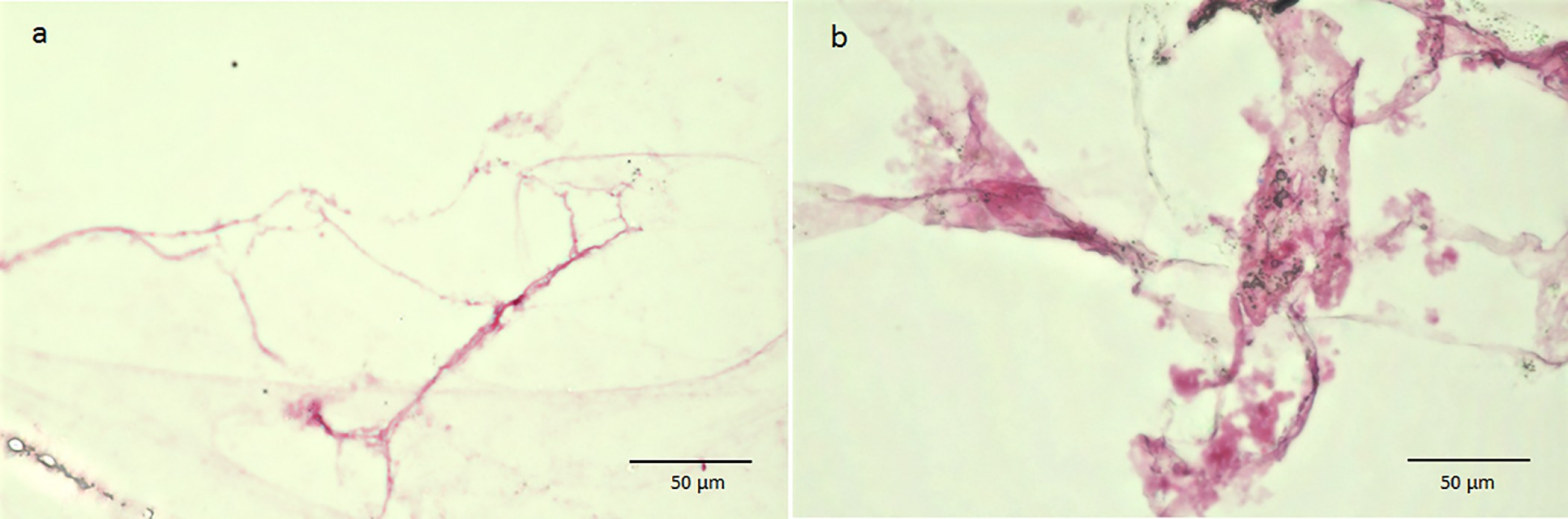
Immunostaining with ER-TR7. The vitreous core is weakly stained, yet the BPM is strongly stained.

In immunostaining with anti-tryptase antibody, the vitreous core was nearly unstained in both cases. However, the BPM region was stained, although weakly (Fig 6). In immunostaining with anti-chymase antibody, all 3 cases also showed a weak staining of the vitreous core, yet strong staining of the BPM. Moreover, in 2 cases, the BPM region showed a membranous structure, which included numerous chymase-positive cells, clearly different from the vitreous core-associated finding (Fig 7). When immunostaining was performed with anti-podoplanin antibody and anti-LYVE-1 antibody (two lymphatic endothelial markers), the BPM was strongly stained, yet the vitreous core was weakly stained (Fig 8). Immunostaining with ER-TR7 (a fibroblast marker antibody) revealed weak staining of the vitreous core, yet strong staining of the BPM (Fig 9).

### Immunofluorostaining of ERM with anti-serine protease antibodies

**Fig 10:**
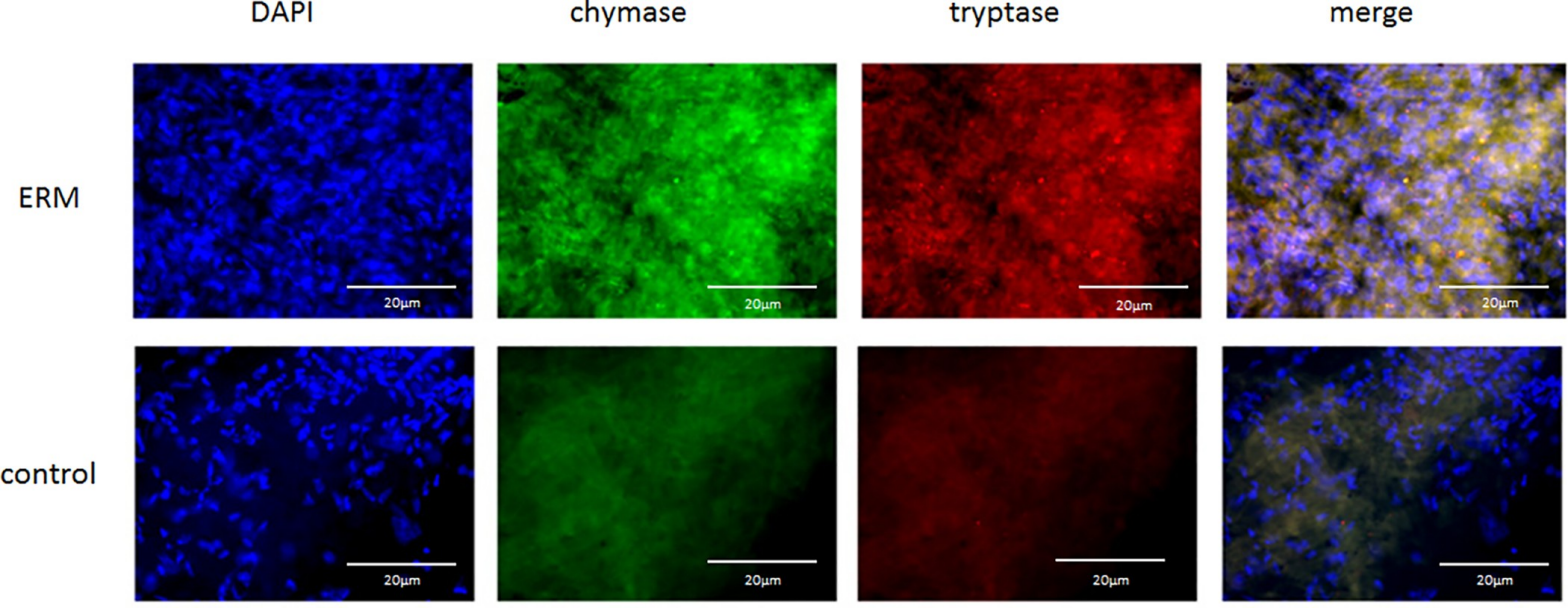
Immunostaining of ERM for tryptase and chymase. The ERM stained weakly positive for both tryptase and chymase, and double-positive cells for tryptase and chymase were sparsely obtained.

The ERM stained weakly positive for both tryptase and chymase, of which positive cells were observed scattered within the ERM (Fig 10). Moreover, these cells were double-stained for both tryptase and chymase, indicating that they are connective-tissue-type mast cells.

## Discussion

Two pioneers of vitreous anatomy, Dr. Jan Worst and Dr. Shoji Kishi, explored the enigmatic structure of the premacular vitreous using different methods [2, 3]. Worst first described the BPM as a liquid-filled sac-like space of the vitreous overlying the macula, and found the BMP connected to the cisternal system, which occupied a significant portion of the vitreous, by injecting India ink into postmortem eyes [12, 13]. Kishi et al observed the vitreous of enucleated eyes by staining with fluorescein, and found that a gel-free region was present in the vitreous anterior to the macula, which they termed PPVP [14]. Worst described the figure of the BPM as dome- or pear-shaped [3]. Kishi et al, on the other hand, reported that the PPVP had a dome-like shape initially, yet expanded into an irregular shape with age, finally occupying more than 50% of the vitreous cavity [2]. Initially, Kishi et al concluded that the PPVP was different from the BPM in several respects as follows. Worst reported that the BPM had a sac-like structure, which may have implied that it is enclosed by a membranous tissue [3, 12, 13]. However, Kishi et al described that the anterior and posterior walls of the borders of the PPVP were the vitreous gel and the PVC, respectively, and that the extent of the PPVP was not necessarily confined to the macular area, as was the case with the BPM [14]. Using contact B-scan ultrasonography, Spaide found an ultrasonographically empty cavity similar to the PPVP in the vitreous, and determined that the mean anteroposterior thickness was 8.4 mm [15].

Eisner first observed the vitreous of autopsy eyes using a slit lamp, and found an optically transparent area overlying the fovea, which he denoted as prefoveal 'Lücke' (i.e., lacuna) [16]. In that study, he reported that the prefoveal Lücke started to develop only after the formation of the fovea had begun, and that the Lücke was not limited to the prefoveal area and almost always extended into the vitreous core as a prefoveal canal. We assume that the PPVP found via the fluorescein staining technique might be consistent with the prefoveal Lücke described by Eisner. In a YouTube video posted on the Internet titled "The vitreous body enigma. A paradigm shift", Worst explained that the cavity located anterior to the BPM was, in fact, Eisner’s Lücke [17].

As has been previously reported, TA is injected into the vitreous cavity to facilitate visualization of the thin premacular membrane during vitrectomy [2]. It is generally considered that this thin membrane, where TA accumulates, is the posterior wall of the PPVP, i.e., namely the PVC. However, Worst et al injected TA directly into this thin premacular tissue during vitrectomy, and depicted the BPM and the connecting cisterns, i.e. the corona petaliformis of Worst. Fine and Spaide [5], as well as Shimada et al [18], also observed that when TA migrated inside the BPM, the cisterns connecting to the BPM were depicted, and that the injected TA could not be readily aspirated without breaking the sac-like structure of the BPM [15]. Based on these findings, we are convinced that the remaining premacular tissue after core vitrectomy is not composed of a single membrane of the PVC, but of a complex tissue consisting of the BPM, connecting cisterns, and the PCV, which adheres to the BPM. Contrary to the general belief, we assume that TA may accumulate on the anterior wall of the BPM, rather than on the posterior surface. The accumulation of TA on the BPM surface might actually occur, presumably because of the feathery texture of the surface, which Worst previously reported [12].

Since the BPM appears to be optically transparent when observed by SD-OCT and SS-OCT, it has been considered that it is a completely empty space without any structure. However, our H&E staining revealed an abundance of cell components in the premacular membrane which supposedly contained the BPM. Thus, our findings indicate that the BPM is not be a lacunar space, but a parenchymal tissue composed of numerous cells.

The findings in several previous studies appear to indicate that the BMP is not merely an empty space. Shimada et al reported that examination via SD-OCT revealed mildly reflective dots inside the BPM [18]. Using a slit lamp, Kakehashi et al discovered that the Tyndall phenomenon was mostly observed in the premacular vitreous, thus indicating that the formed vitreous gel was present in the BPM [19]. Sebag and Balazs reported observing a large bundle of prominent fibers coursing anteroposteriorly and entering the retrohyaloid space via the premacular hole in a vitreous specimen [20]. Using electron microscopy, Jongebloed et al reportedly observed the BPM displayed as a collagenous structure that was denser than other regions [21]. The fact that fluorescein staining of the posterior wall of the PPVP, which we firmly believe is the BMP adhered on the PVC, appears to also support our assumption that the BPM is a gel-like parenchymal tissue.

In an extension of our research regarding the relationship between the renin-angiotensin system and diabetic retinopathy [22, 23], we found that in comparison with other diseases, tryptase activity is higher in the vitreous of ERM and MH, and that chymase activity is higher in the vitreous of MH [10, 11]. Based on those findings, we speculated that these serine proteases might be involved in the pathogenesis of ERM and MH. In this present study, we repeated the measurement of tryptase and chymase activities in four vitreoretinal diseases, and obtained results that are nearly identical to those in our previous studies [10, 11]. No significant differences were identified in serum tryptase levels among the four diseases, and serum chymase activity did not exceed the detection limit in each of the diseases. Such undetectable levels of chymase were attributable to the presence of serine protease inhibitors (serpins) in the serum. It was previously reported that serpins strongly inhibit serum chymase activity, thereby causing a complete loss of the activity within seconds after chymase enters the serum [24]. Moreover, both tryptase and chymase are reportedly unable to pass through the blood-brain barrier, thus indicating that serine proteinases in the vitreous are produced in intraocular tissues [25].

Mast cells produce tryptase, chymase, and other bioactive substances, such as histamine and tumor necrosis factor-alpha, which have attracted attention in recent years due to the reports of their involvement in fibrosis, apoptosis, and the remodeling of various tissues [26–28]. In the eye, mast cells are reportedly present in the choroid, the ciliary body, the conjunctiva, and the sclera [29]. As far as we know, tissue resident mast cells have not been reported in the vitreous, except in cases of Eales disease and persistent hyperplastic vitreous [30, 31]. Accordingly, the intraocular tissue where the mast cells produced tryptase and chymase in the vitreous had previously remained unknown. However, the findings in this present study revealed that numerous tryptase and/or chymase positive cells are present in the BPM, and that cytoplasmic staining with toluidine blue revealed a somewhat metachromatic staining in the BPM. The ERM samples were also partially stained by anti-tryptase and anti-chymase antibodies. These results indicate that the origins of serine proteinases in the vitreous of ERM and MH are the mast cells in the BPM and the ERM samples.

It should be noted that there are two types of mast cells, *i*.*e*., tryptase-positive mucosal-type mast cells and tryptase/chymase-positive connective-tissue-type mast cells. The findings in our previous studies showed that compared with other diseases, tryptase activity was higher in ERM and that chymase activity was higher in MH [10, 11], thus indicating that mucosal-type and connective tissue-type mast cells may have been predominant in the BPM of ERM and MH, respectively. Since it has been reported that tryptase can induce a profibrotic response [32], while chymase can induce a proapoptotic response [33, 34], our results seemed to well explain the pathogenesis of ERM and MH.

Previous reports showed that vitreous cell components were mostly hyalocytes and fibroblasts. Hyalocytes are reportedly located at highest concentration in the vitreous cortex, especially in the vitreous base and in the posterior pole [35, 36]. Fibroblasts, which constitute less than 10% of the vitreous cells, are reportedly commonly present at the vitreous base and near the optic disc [35, 37]. In this study we performed immunostaining of the premacular membrane, using antibody against ER-TR7, a marker of reticular fibroblasts, and found that reticular fibroblasts besides mast cells may have resided in the BPM.

Mast cells are derived from pluripotent hematopoietic stem cells in bone marrow, and migrate hematogenously or lymphogenously as undifferentiated cells to each tissue, where they proliferate and differentiate [38, 39]. Studies in recent years have revealed that mast cells express c-kit and C-X-C chemokine receptor type 4 (CXCR4), of which the ligands are stem cell factor (SCF) and C-X-C motif chemokine 12 (CXCL12) [also known as stromal cell-derived factor 1 (SDF-1)], respectively [40–42]. SCF promotes the proliferation, migration, and differentiation of mast cells, and CXCL12 induces the migration of mast cells [43–48]. Since fibroblasts secrete SCF and CXCL12, it is postulated that fibroblasts in the BPM may lead to accumulation and proliferation of mast cells, thereby providing a source of serine proteases in the vitreous of macular diseases. Since the premacular vitreous is constantly exposed to light, it may promote the photoaging of mast cells and fibroblasts, the same is in the skin [49–54]. We assume that age-related onset of ERM and MH could be explained by a dysfunction of mast cells and fibroblasts in the BPM due to aging.

As described above, the immunostaining of the BPM clearly showed more intense staining for anti-tryptase and anti-chymase antibodies than the vitreous core. However, positive staining for these serine proteases was observed not just in the cells, but also in the surrounding tissues. Since serine proteases have the property to deposit in the extracellular matrix, our results may indicate tryptase and chymase are adsorbed on collagen fibers in the vitreous.

The physiological roles of the BPM have yet to be fully elucidated. However, it has recently been reported that retinal stem cells are present in the peripheral retina and the ciliary body [55, 56]. We previously reported that the photoreceptor layer of the foveola and Henle’s layer were positive for nestin, a neural stem cell marker, in monkey eyes, and proposed that stem cell-like cells are probably present in the foveola of primates [57]. Fibroblasts are known to constitute the stem cell niche of intestinal crypts. Mast cells are also reported to be present near the intestinal stem cells, and affect their functions [58–60]. Thus, fibroblasts and mast cells residing in the BPM might provide a microenvironment for the undifferentiated cells in the foveola. Meanwhiler, Rossi et al proposed via computer simulation that the BPM protected the fovea from shear stress caused by the movement of the vitreous [61], and Worstpostulated that the BPM has a protective function for the macula in hydrodynamic and biochemical senses [12, 62]. Eisner first proposed that intravitreal tracts regulated the intravitreal fluid transports [16]. This was followed by Worst and Los advocating that the cisternal system including the BPM is involved in the transport of intravitreal fluid and excretes the macular fluid to the anterior portion of the vitreous [12].

The human body has two circulatory systems that transport body fluids, the cardiovascular system and the lymphatic system. Although the blood capillaries carry the red-colored blood, and are tubular structures, the lymphatic capillaries carry the clear watery fluid called lymph, and are irregularly shaped. Therefore, we assume the cisternal systems in the vitreous are similar to the lymphatic system, both functionally and morphologically. Especially, the subperitoneal lymphatic lacuna in the diaphragm is unique for its large size and multiple morphology, and can be recognized by its broad, flattened enlargement and blinding ends of lymphatic vessels, which communicate with each other to form a rich plexus [63]. We consider that the morphology of the lymphatic lacuna highly resembles the appearance of the cisternal system, which Worst and Los investigated using the ink injection method for a long-term period [3, 12, 62].

Los reported that in the vitreous of children, the BPM and the area of Martegiani are completely separated and are limited in the premacular and preoptic areas, respectively, and that these sac-like structures expand and connect to each other with age, ultimately reaching the anterior parts of the vitreous [64]. As mentioned above, Eisner and Kishi also observed a similar phenomena [2]. Los advocated that the age-related increase of the volume of the cisternal system would be consistent with a differentiation process rather than matrix degeneration, namely, syneresis of the vitreous gel [64].

Anatomically and developmentally, the retina is known as an extension of the brain, and the vitreous is regarded as corresponding to the pia mater. The central nervous system had previously been considered an organ devoid of lymphatic vessels. However, it has recently been discovered that functional lymphatic vessels line the dural sinuses and that the pia mater appears to possess a pre-lymphatic capillary system, and are thus involved in lymphatic drainage [65, 66]. In those studies, it was reported that the meningeal lymphatic network develops postnatally, as does the cisternal system in the vitreous, and that mast cells, that we found in the premacular membrane, are abundantly present in the pia mater, especially in the pia mater overlying the cerebellum and thalamus.

Based on the hypothesis proposed by Worst, we theorize that the cisternal system of the vitreous may have a function to excrete intraocular fluids out of the eye. Intraocular pressure reportedly has a tendency to increase after vitrectomy [67], presumably due to the destruction of the cisternal system acting as the drainage tracts of intraocular fluids. Recently, it was reported that LYVE-1, a lymphatic endothelial cell-specific marker, is expressed in the cells of fetal vitreous [68]. With these findings in mind, we performed immunostaining of the vitreous samples with antibodies against LYVE-1 and podoplanin, lymphatic endothelial cell markers, which resulted in strong staining in the BPM region.

Unlike lymphatic vessels, lymph nodes are a parenchymal tissue [69]. As stated above, we found that in the premacular membrane, presumably containing the BPM, numerous cells exist, indicating that the BPM was also a parenchymal tissue, the same as lymph nodes. Mast cells and reticular fibroblasts, which we found in the premacular membrane, reportedly reside in lymph nodes and act physiologically [70–72]. As described above, Kishi et al reported that elasticity was higher in the premacular tissue than in other parts of the vitreous [2]. Worst mentioned that the BPM had a different texture and is expandable. Similarly, lymph nodes are elastic, and swell up during an infection [73, 74]. While it is well known that bacteria and viruses trapped in lymph nodes, Jongebloed et al observed that some bacteria is trapped in the BPM using scanning electron microscopy [24]. By observing the lymph nodes using OCT, they were depicted as a hyporeflective area, as was the BPM. Based on the above findings, we speculate that the BPM has very similar properties to lymph nodes, except that there is a distribution of blood vessels within lymph nodes.

In the previous studies by Worst and Los, and Worst, the authors described the BPM as being a liquid-filled space, probably because the injected India ink filled the space [12, 13]. However, lymph fluids are able to pass through lymph nodes, which are a parenchymal tissue. Moreover, lymphatic vessels and lymph nodes are usually observed by staining with India ink [12]. Therefore, it is possible that the BPM is a parenchymal tissue, similar to lymph nodes, although liquids easily pass through it.

It should be noted that this present study did have some limitations. First, the vitreous samples that we collected may have contained different parts of the cisternal system. Second, the vitreous core samples probably contained samples from outside of the cisternal system. Thus, we presumed that this is the reason why the appearance of the immunostaining was extremely different among the vitreous core samples. Third, the BPM samples would have contained corona petaliformis of Worst and the area of Martegiani, not just the BPM. Nevertheless, our immunohistological analysis findings demonstrated that the premature membrane including the BPM would be a gel-like parenchymal tissue containing an abundance of cells, rather than a fluid-filled empty space. In addition, our findings also indicate that mast cells in the BPM may be a source of tryptase and chymase in the vitreous, which are presumably involved in the onset and progression of ERM and MH.

In conclusion, while further studies are needed, we hope that the findings in this study will provide a foothold for the elucidation of the anatomy of the premacular vitreous and pathogenesis of macular diseases.

## Supporting information

S1 Fig Low power magnification original data. (PPTX)

S1 File Chymase activities in the vitreous body and serum (original data). (XLSX)

S2 File Tryptase activities in the vitreous body and serum (original data). (XLSX)
