# Supplementary figures and images for: Involvement of premacular mast cells in the pathogenesis of macular diseases
Source: PLoS One. 2019 Feb 22;14(2):e0211438. doi: 10.1371/journal.pone.0211438 (PMC6386310; doi:10.1371/journal.pone.0211438)

## Slide 1
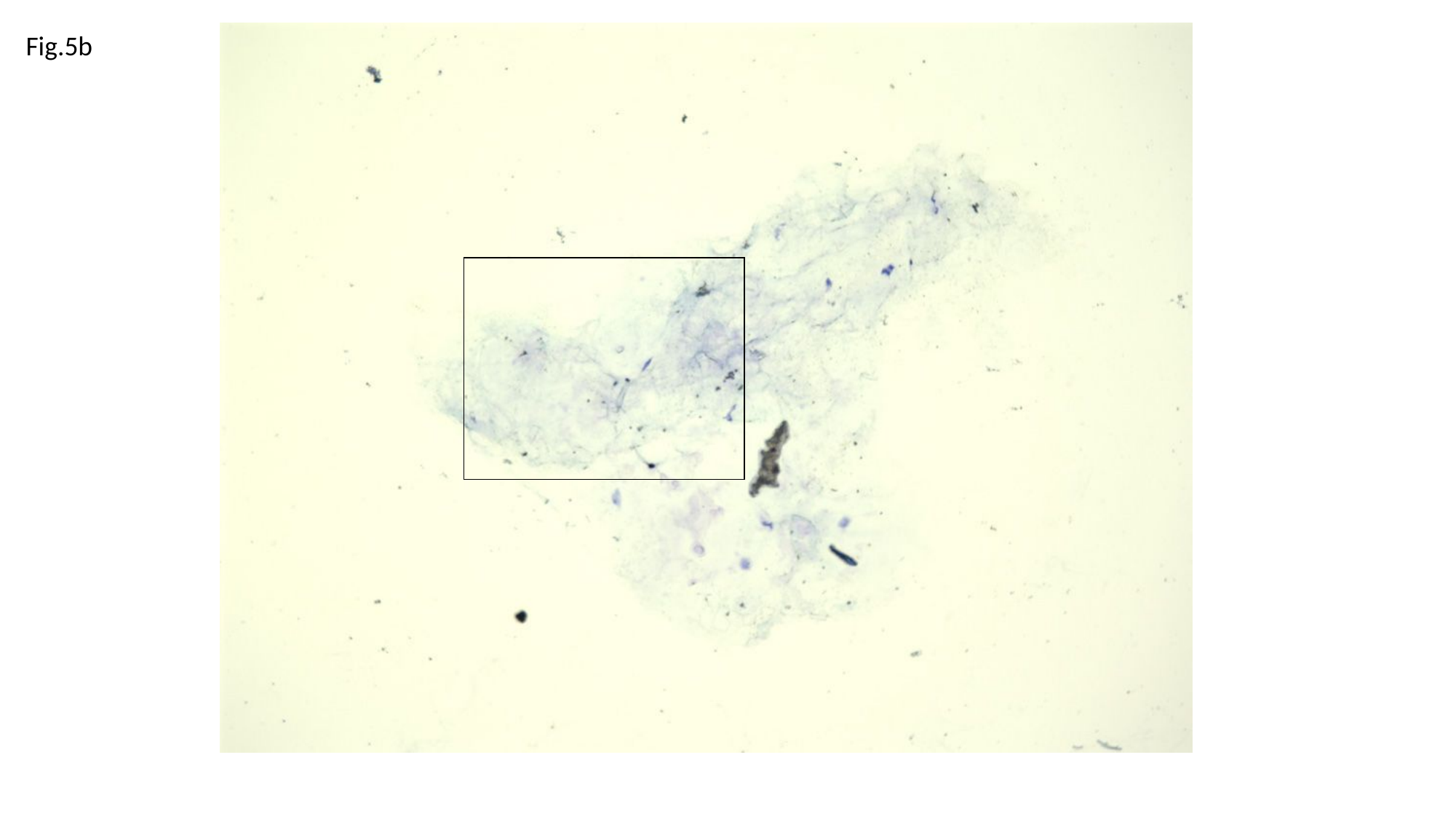

Fig.5b

## Slide 2
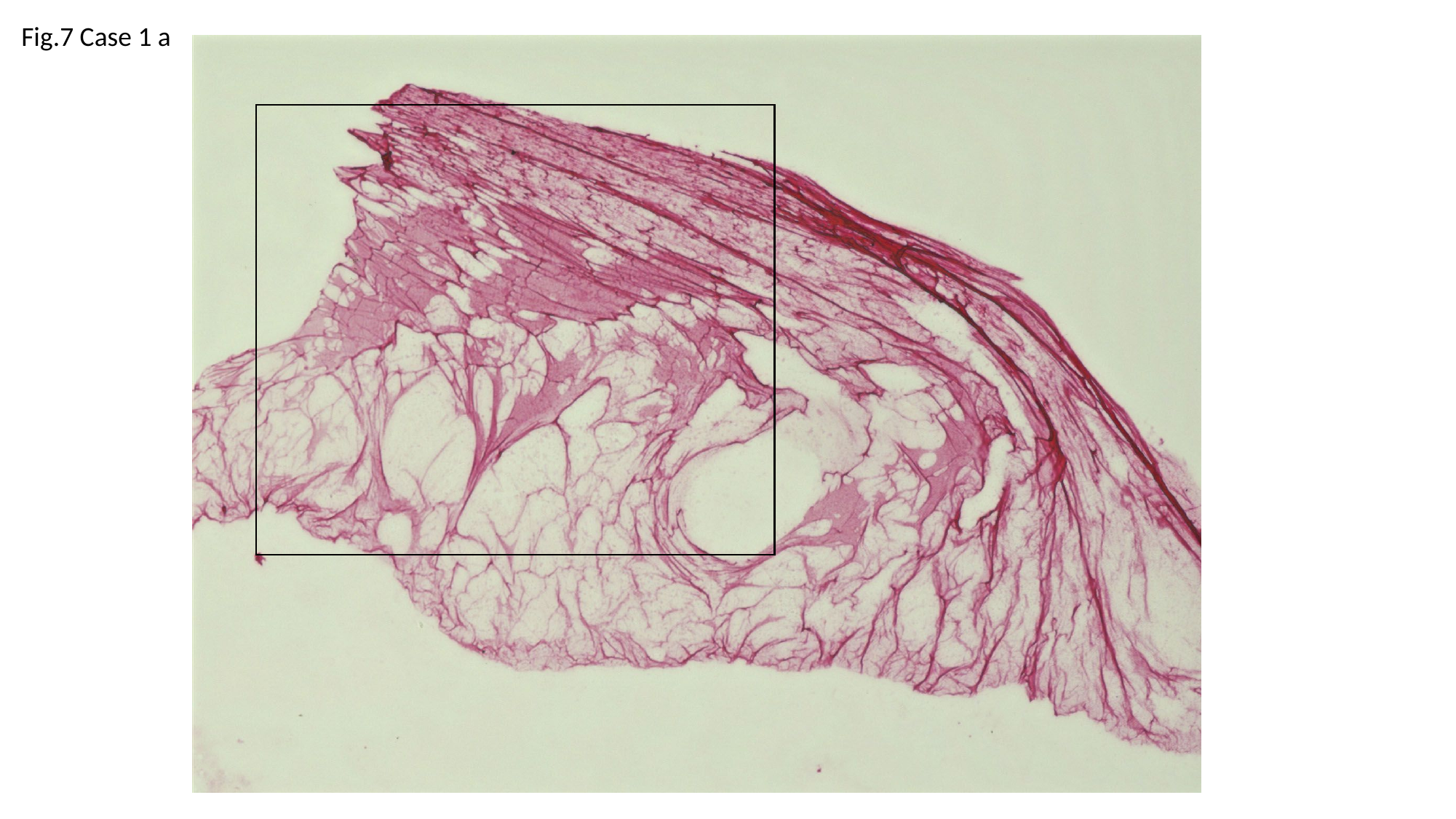

Fig.7 Case 1 a

## Slide 3
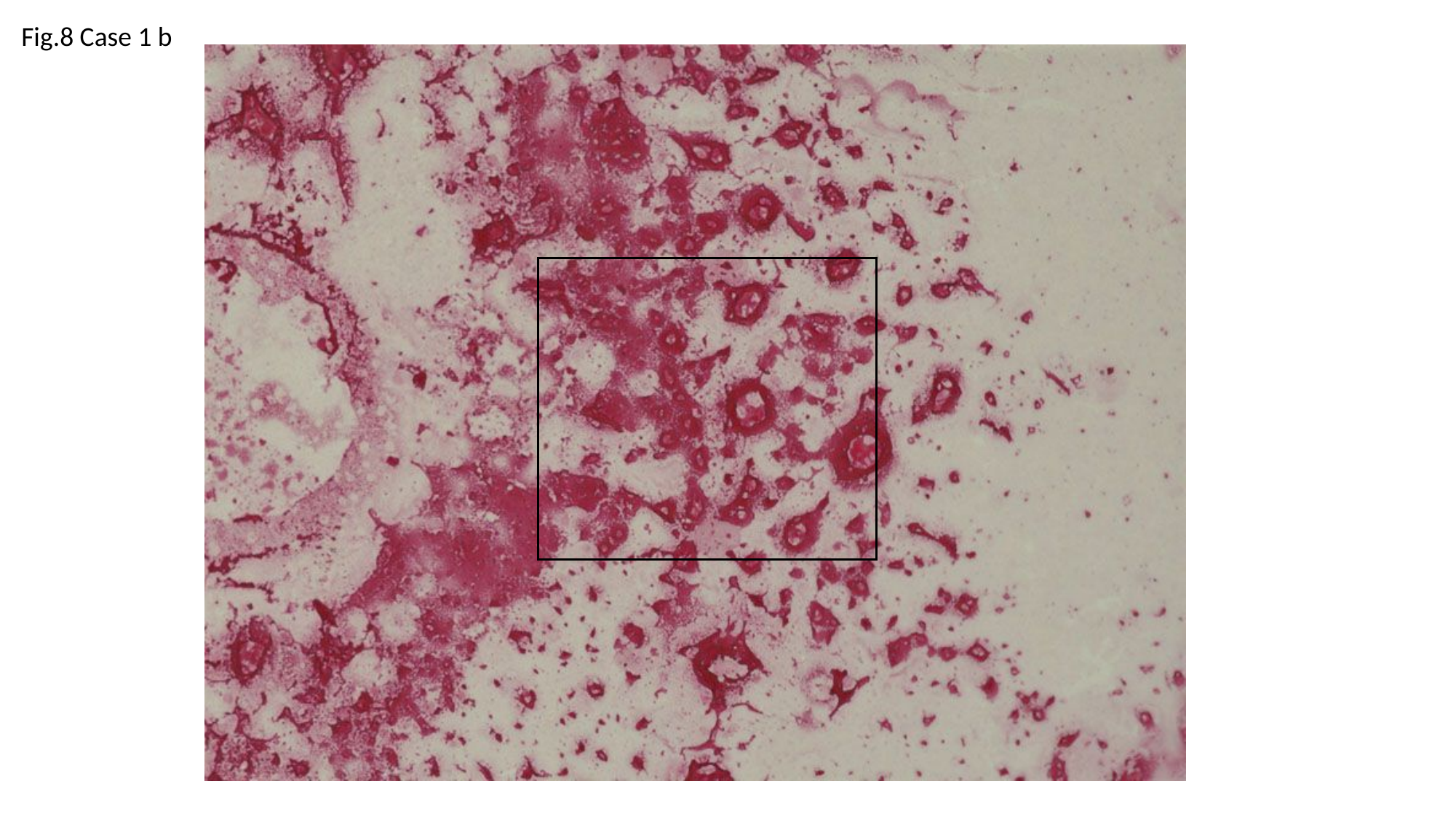

Fig.8 Case 1 b

## Slide 4
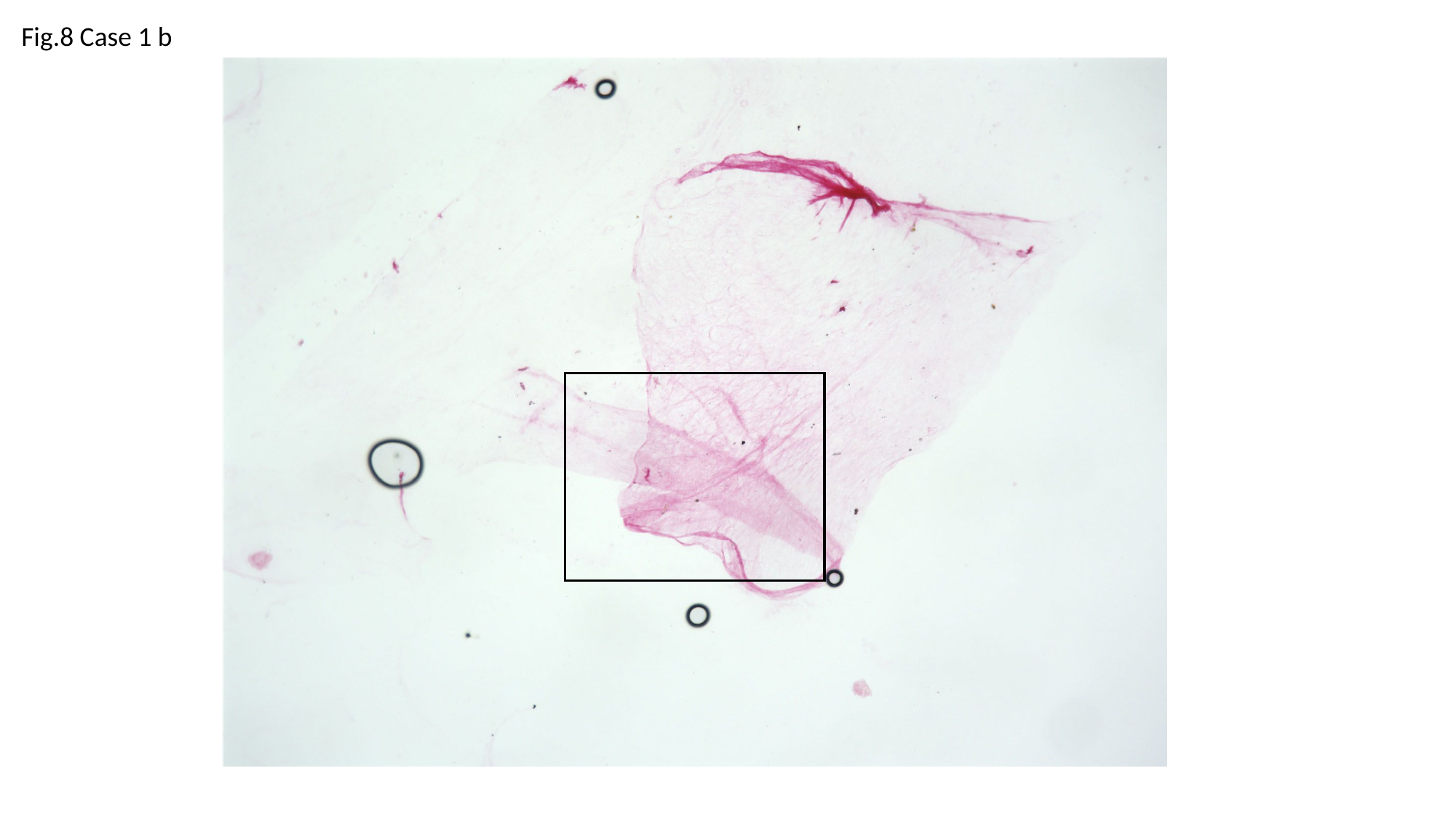

Fig.8 Case 1 b

## Slide 5
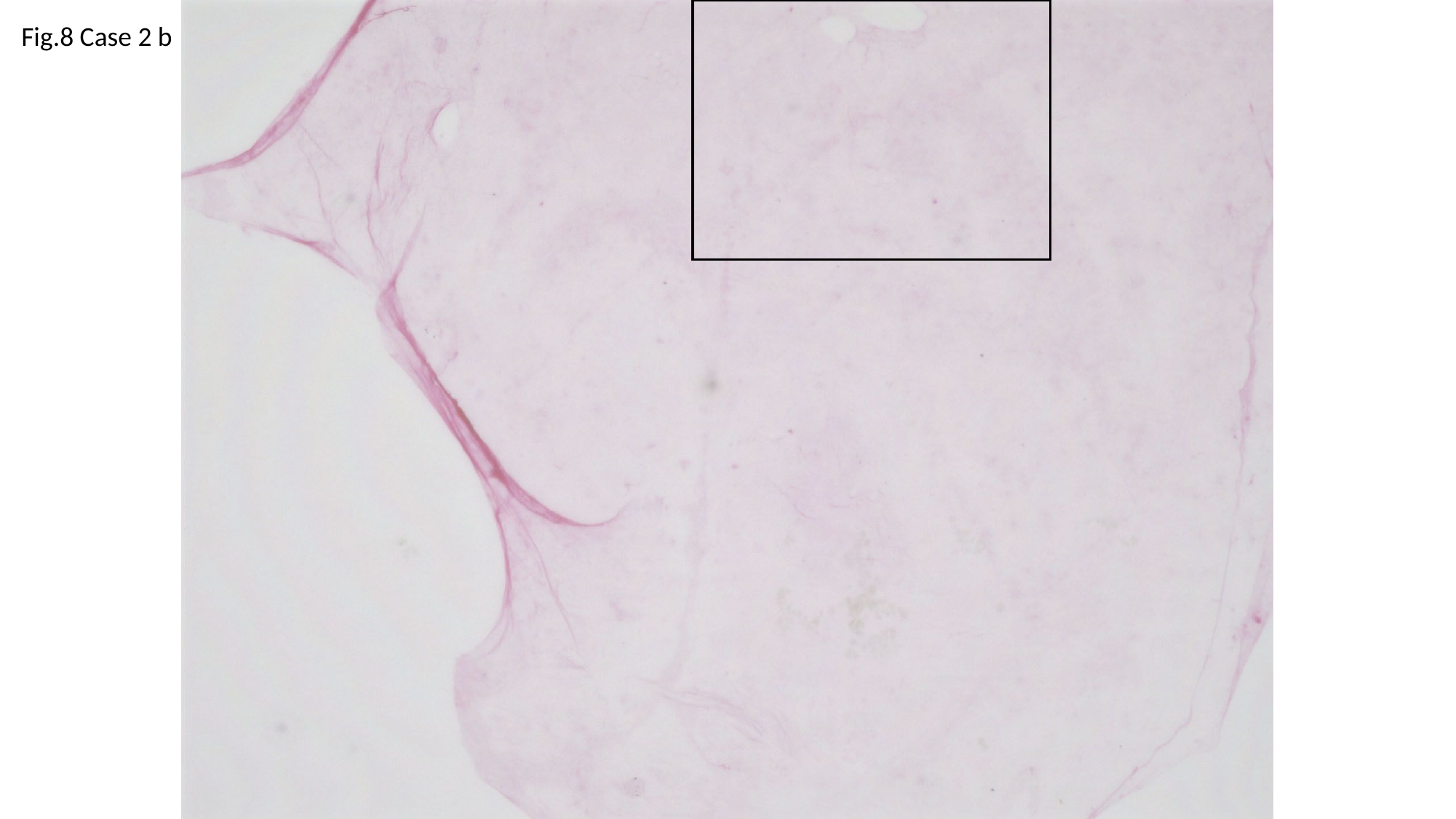

Fig.8 Case 2 b

Supplement: S1 Fig — (PPTX) [file pone.0211438.s001.pptx]
